# A scoping review of the methodological approaches used in retrospective chart reviews to validate adverse event rates in administrative data

**DOI:** 10.1093/intqhc/mzae037

**Published:** 2024-04-24

**Authors:** Anna Connolly, Marcia Kirwan, Anne Matthews

**Affiliations:** School of Nursing, Psychotherapy and Community Health, Dublin City University, Dublin D09 V209, Ireland; School of Nursing, Psychotherapy and Community Health, Dublin City University, Dublin D09 V209, Ireland; School of Nursing, Psychotherapy and Community Health, Dublin City University, Dublin D09 V209, Ireland

**Keywords:** patient safety, healthcare quality, adverse events, administrative data, retrospective chart review

## Abstract

Patient safety is a key quality issue for health systems. Healthcare acquired adverse events (AEs) compromise safety and quality; therefore, their reporting and monitoring is a patient safety priority. Although administrative datasets are potentially efficient tools for monitoring rates of AEs, concerns remain over the accuracy of their data. Chart review validation studies are required to explore the potential of administrative data to inform research and health policy. This review aims to present an overview of the methodological approaches and strategies used to validate rates of AEs in administrative data through chart review. This review was conducted in line with the Joanna Briggs Institute methodological framework for scoping reviews. Through database searches, 1054 sources were identified, imported into Covidence, and screened against the inclusion criteria. Articles that validated rates of AEs in administrative data through chart review were included. Data were extracted, exported to Microsoft Excel, arranged into a charting table, and presented in a tabular and descriptive format. Fifty-six studies were included. Most sources reported on surgical AEs; however, other medical specialties were also explored. Chart reviews were used in all studies; however, few agreed on terminology for the study design. Various methodological approaches and sampling strategies were used. Some studies used the Global Trigger Tool, a two-stage chart review method, whilst others used alternative single-, two-stage, or unclear approaches. The sources used samples of flagged charts (*n* = 24), flagged and random charts (*n* = 11), and random charts (*n* = 21). Most studies reported poor or moderate accuracy of AE rates. Some studies reported good accuracy of AE recording which highlights the potential of using administrative data for research purposes. This review highlights the potential for administrative data to provide information on AE rates and improve patient safety and healthcare quality. Nonetheless, further work is warranted to ensure that administrative data are accurate. The variation of methodological approaches taken, and sampling techniques used demonstrate a lack of consensus on best practice; therefore, further clarity and consensus are necessary to develop a more systematic approach to chart reviewing.

## Introduction

Patient safety is a priority for health systems with 1 in 10 patients experiencing an in-hospital adverse event (AE) in high-income countries annually. The cost of patient harm is estimated at 1–2 trillion US Dollars per year [1], arising from additional resources, increased care, and prolonged hospital stays [2].

Accurate AE data are fundamental to addressing these costs and enabling patient safety and healthcare quality advancements [1]. Administrative healthcare data are a valuable source of information on AEs and a potentially efficient tool for monitoring patient safety [3]. Translating chart data into alphanumeric data [4] provides a coded summary of the patient and their encounter with the health system [5]. The International Classification of Diseases (ICD), used for this purpose by all World Health Organization member states, provides a standardized method of reporting and monitoring health-related issues across hospitals, regions, and countries [6].

Medical knowledge advancements have resulted in many ICD revisions [7]. Various derived classifications of ICD and ICD modifications have been developed to monitor conditions and complications in specific areas or settings [8] and address country-specific needs [9].

Administrative data-based AE detection tools have been developed to screen for potential AEs and assess their preventability [10]. The Agency for Healthcare Research and Quality (AHRQ) Patient Safety Indicators (PSIs) demonstrate the potential use of administrative data for benchmarking rates of AEs across the USA [11]. Similarly, the Classification of Hospital-acquired Diagnoses (CHADx) in Australia [12] generates patient safety data for monitoring AEs. The increasing popularity of administrative data-based safety indicators has coincided with an increase in validation studies [10].

Inaccurate and poor recording of AEs [13, 14] warrants validation of administrative datasets and ICD coding [15]. A systematic and standardized approach to measuring AEs in administrative data would improve dataset accuracy and enable benchmarking across hospitals [16] for comparison and improvement [17].

Whether a single gold standard for the measurement of harms exists is disputed [18, 19]; however, medical charts are often considered a ‘gold standard’ source of patient information [20–22]. Despite their limitations, chart reviews are commonly used to assess healthcare quality and inpatient care [23] and identify whether administrative data accurately reflect the events of a patient’s hospitalization [24]. They utilize readily available data and are more feasible than observational studies which are time-consuming, expensive, and complex in terms of confidentiality and bias [25]. The Harvard Medical Practice Study (HMPS) and the Global Trigger Tool (GTT) are examples of two-stage chart review approaches [26]. Nonetheless, validated administrative data are potentially another valuable source of AE data [3, 27–29]. The need to understand how chart reviews have been conducted to identify optimal practices for measuring administrative data accuracy provides rationale for this review.

## Methods

This review was conducted in line with the Joanna Briggs Institute (JBI) methodology for scoping reviews [30]. A protocol for this study has previously been published [31]. Searches in PubMed (MEDLINE), CINAHL, the JBI Evidence Synthesis, and The Cochrane Database of Systematic Reviews did not identify any similar scoping or systematic reviews.

### Objectives

1. To present an overview of chart reviewing approaches and tools used to validate rates of AEs in administrative datasets.
2. To collate and map evidence of chart reviewing to measure the reliability of these datasets.

#### Inclusion and exclusion criteria

The inclusion and exclusion criteria are outlined in Table 1.

**Table 1.** Inclusion and exclusion criteria.

	Inclusion criteria	Exclusion criteria
Population	Hospital inpatients whose medical chart information has been extracted, assigned ICD codes, and recorded in administrative data	Any population other than hospital inpatients
Concept	The validation of the accuracy of rates of AEs in administrative data and the methodological approaches used to conduct retrospective chart review	Sources that do not validate rates of adverse events in administrative data through retrospective chart review
Context	Studies that validated data collected within hospital-based settings	Studies reporting on settings other than hospital-based settings
Types of evidence sources	All sources of evidence other than review articles were included as this review explored the specific methods used to validate rates of AEs in administrative data through patient chart review. Referring to primary research articles only ensured that credit was accurately assigned to each author of the included sources [84]	Sources using AI or subsets of AI such as Natural Language Processing (NLP) to conduct chart reviews to validate rates of adverse events in administrative data

### Changes since protocol

The exclusion of review articles is a deviation from the study protocol which initially stated that the types of evidence sources would be left open. All available articles were included by searching reference lists of any identified reviews.

### Search strategy

Searches in PubMed (MEDLINE) and CINAHL databases, conducted with a university librarian, yielded titles and abstracts from which text words were extracted and used to develop a search strategy (Supplementary material). Using this strategy, PubMed (MEDLINE) and CINAHL were searched on 4 April 2023 and Web of Science and Scopus were searched on 24 April 2023. The reference lists of included sources were searched and a search for grey literature was conducted [32].

The HMPS was the first large-scale medical record review conducted over 30 years ago [33]; therefore, literature published in English between 1991 and 2023 was eligible for inclusion.

### Source of evidence screening and selection

**Figure 1:**
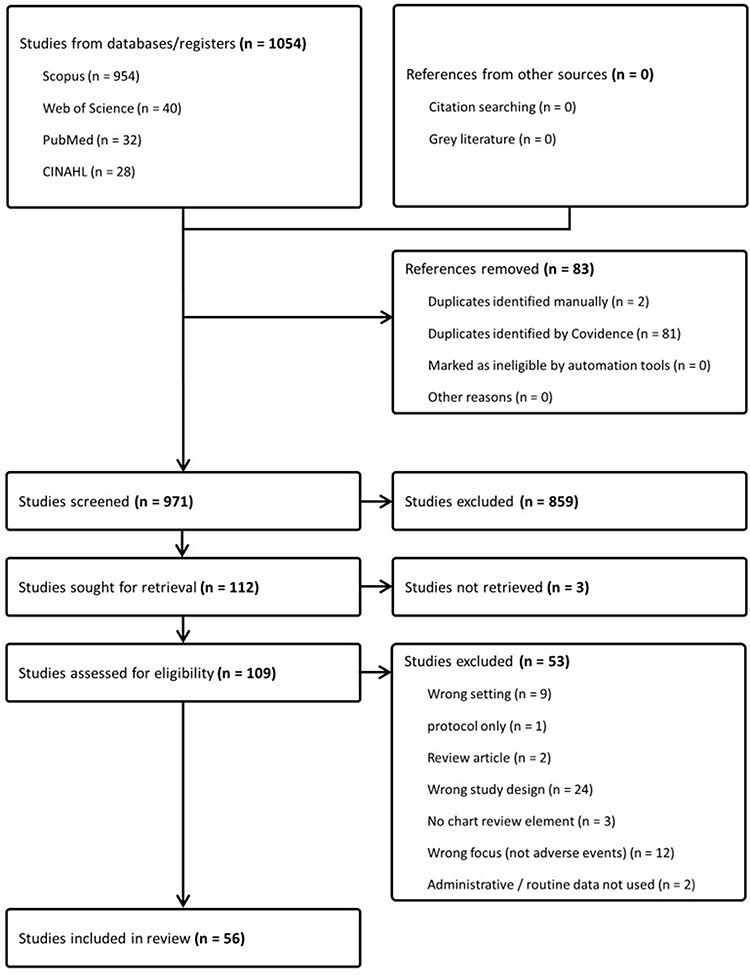
PRISMA flowchart of source selection process

Two researchers independently screened study titles and abstracts. Full-text versions of potentially relevant sources were assessed against the inclusion criteria. Excluded sources were assigned an exclusion reason (Fig. 1). Conflicts were resolved through discussion and included sources were confirmed. A third researcher consulted on certain sources.

### Data extraction

A data extraction template was developed and piloted. Key variables included: study title, name of author(s), year of publication, country, study aim, study design, methods, study population, specialty associated with the AE reported on, and key findings relating to the accuracy of the administrative data and administrative data-based detection tools.

### Analysis and presentation of results

The extracted data were exported and arranged into a charting table in Microsoft Excel that was continuously updated. A descriptive numerical summary and a qualitative content analysis of the included studies were conducted.

The accuracy of the administrative data or administrative data-based detected tools was determined based on the specificity, sensitivity, positive predictive value (PPV) or negative predictive value (NPV), where reported, and on statements from the authors of each study relating to the validity of the datasets or detection tools and the extent to which they can be used to accurately measure rates of AEs.

## Results

### Search results

Following duplicate removal and screening of the search results as outlined in the Preferred Reporting Items for Systematic reviews and Meta-Analyses flowchart (Fig. 1), 56 sources were included. A qualitative content analysis of the literature was conducted, and study findings are presented in a tabular and descriptive format.

### Demographics and study characteristics

Studies by country, publication year, specialty, and study design are outlined in Table 2. All 56 studies were published between 1997 and 2022 and were conducted in 12 countries with 31 (55%) in the USA. Various specialties were explored; however, 19 studies reported on AEs in surgical specialties and 14 studies validated AEs in all specialties. All studies included a retrospective chart review; however, little consistency in relation to the methods and study design was identified. Of the included studies, 32 measured the accuracy of the administrative data coding and 24 reported on the accuracy of the administrative data-based AE detection tools (Table 2).

**Table 2.** Distribution of included sources according to country, year of publication, clinical specialty associated with the adverse event(s), named study design, methodological approach, and sampling strategy.

Country in which the study conducted	No. of studies	Reference number
USA	31	[34, 63–65, 67, 68, 74, 79, 82, 85–106]
Australia	5	[35, 69, 77, 107, 108]
Canada	6	[76, 83, 109–112]
Belgium	1	[113]
Germany	4	[22, 78, 81, 114]
Italy	2	[75, 115]
Japan	1	[116]
New Zealand	1	[80]
Norway	1	[117]
Sweden	2	[118, 119]
Switzerland	1	[120]
Taiwan	1	[66]
**Year of publication**	**No. of studies**	**Reference number**
1997–2002	2	[90, 106]
2003–2007	2	[91, 99]
2008–2012	11	[34, 63, 64, 67, 69, 74, 75, 92, 97, 101, 113]
2013–2017	20	[22, 65, 68, 76–78, 85–87, 89, 93, 94, 98, 100, 102–105, 111, 112]
2018–2022	21	[35, 66, 79–83, 88, 95, 96, 107–110, 114–120]
**Clinical speciality**	**No. of studies**	**Reference number**
All specialities	14	[22, 78, 80, 81, 91, 93, 97, 106, 107, 109, 111–113, 118]
Surgical	19	[34, 35, 64, 67, 68, 74, 75, 83, 86, 87, 92, 96, 100, 102–104, 110, 115, 117]
Paediatrics	4	[63, 77, 88, 99]
Oncology	4	[85, 94, 108, 116]
Pharmacology	4	[65, 66, 79, 114]
Obstetrics	4	[69, 89, 101, 120]
Geriatrics	1	[76]
Orthopaedics	1	[119]
Medical	1	[90]
Medical/surgical/trauma/ED	4	[82, 95, 98, 105]
**Named study design**	**No. of studies**	**Reference number**
Chart review study	9	[35, 63, 68, 77, 94, 96, 105, 112, 120]
Retrospective chart review study	10	[67, 89, 91–93, 98, 99, 101, 107, 113]
Retrospective chart review validation study	6	[65, 76, 79, 83, 114, 116]
Retrospective cross-sectional study	6	[64, 74, 82, 97, 100, 102]
Other	25	[22, 34, 66, 69, 75, 78, 80, 81, 85–88, 90, 95, 103, 104, 106, 108–111, 115, 117–119]
**Methodological approach**	**No. of studies**	**Reference number**
Single stage review	39	[22, 34, 35, 67, 68, 74, 76–78, 80–83, 85–87, 89–94, 97–99, 103, 104, 107–116, 118, 120]
Two stage review	7	[65, 66, 75, 79, 88, 95, 106]
Global Trigger Tool (GTT)	3	[105, 117, 119]
Not clear	7	[63, 64, 69, 96, 100–102]
**Sampling strategy**	**No. of studies**	**Reference number**
Flagged and random	11	[75, 76, 82, 91, 93, 100, 102, 106, 107, 113, 115]
Random	21	[22, 34, 35, 69, 78, 83, 85, 89, 90, 94–96, 103, 105, 109, 110, 116–120]
Flagged	24	[63–68, 74, 77, 79–81, 86–88, 92, 97–99, 101, 104, 108, 111, 112, 114]
**Validation of administrative data or administrative data-based AE detection tools**	**No. of studies**	**Reference number**
Administrative data coding	32	[34, 35, 65, 66, 69, 76–83, 85, 87, 88, 90, 91, 94–96, 98, 103, 108–111, 113, 114, 116, 117, 120]
Administrative data-based AE detection tool	24	[22, 63, 64, 67, 68, 74, 75, 86, 89, 92, 93, 97, 99–102, 104–107, 112, 115, 118, 119]

Other notable study features include coding classification system, administrative data-based detection tool, type of medical record system used, and number of medical records reviewed (Table 3). The accuracy of the AHRQ PSIs was measured in 16 studies. Excluding five studies that were unclear in relation to the coding classification system used, all other administrative data-based AE detection tools used versions of ICD.

**Table 3.** Key study features of included studies—author, coding classification system, administrative data-based detection tool, type of medical record system used, and number of medical records reviewed.

First author and year of publication	Coding classification system used	Patient safety data system	Type of record system	Number of medical records reviewed
Ackroyd-Stolarz 2014	ICD-10-CA		Not clear	300
Amelung 2022	ICD-10		Not clear	69
Ammann 2017	ICD-9 CM		Charts stored on password-protected server	131
Ammann 2018	ICD-9 CM		Not clear	75
Awad 2015	ICD-9		Electronic medical records	355
Bensley 2013	ICD-9		Not clear	1342
Borzecki 2011	ICD-9 CM	AHRQ PSI	Electronic medical records—VA (veterans administration) EMR data were accessed using VistAWeb, a program enabling centralized access to EMR data from all VA facilities	119
Borzecki 2013	ICD-9 CM		Electronic medical records—VA (veterans administration) EMR data were accessed using VistAWeb, a program enabling centralized access to EMR data from all VA facilities	125
Caminiti 2012	ICD-9	AE detection system developed by the authors	Not clear	504
Campbell 2011	ICD-9		Hospital patient charts and electronic medical records	100
Chen 2013	ICD-9 CM	AHRQ PSIs	Electronic medical records—VA (veterans administration) EMR data were accessed using VistAWeb, a program enabling centralized access to EMR data from all VA facilities	224
Cheng 2021	ICD-10 CM		Not clear	1384
Duke 2022	Not clear	The national hospital-acquired complication programme developed by the Australian Commission on Safetyand Quality in Healthcare (ACSQHC)	Millenium™ (Cerner Corp., Kansas City, MI, USA) electronic medical records system	722 (462 not flagged and 260 flagged)
Eastwood 2022	ICD-11 and ICD-10 CM		Not clear	1009
Eisler 2018	ICD-9		Electronic medical records—hospital Clinical Data Warehouse houses all hospital administrative data and electronic health records	254
Foglia 2015	ICD-9 CM	Adverse outcome index: an obstetrical quality measure	Electronic medical records	4252
Fujiwara 2022	ICD-10		Electronic medical records—EMRs from Kurashiki Central Hospital, Japan, as the reference standard	641 cases
Geraci 1997	ICD-9 CM		Not clear	1837
Graham 2019	ICD-10-CA		Paper-based and electronic medical records	154
Guzzo 2019	ICD-9 CM	AHRQ PSIs	Not clear—the clinical records for data extraction were provided by the Health Hospital Authority	
Ho 2017	ICD-10-CA		Not clear—nursing consult documentation	1217
Hougland 2006	ICD-9 CM		Not clear	1961—random and 1142 flagged
Kaafarani 2011	ICD-9 CM	AHRQ PSI	Electronic medical records—VA (veterans administration) EMR data	336
Kandel 2021	ICD-10		Not clear	8844
Kuklik 2019	ICD-10 GM		Electronic and paper-based charts	2326 cases
Kuklik 2017	ICD-10		Electronic and paper-based charts	807 cases for PPV assessment and 1510 charts for sensitivity assessment
Maass 2015	ICD-10 GM	AHRQ PSIs, OECD PSI, Author-developed PSIs	Paper-based charts	3000 cases
Magnéli 2019	ICD-10	Instrument based on a set of 13 specific ICD codes and one code category (I-codes: diseases of the circulatory system) defining AEs.	Medical records were obtained as paper copies or were reviewed on location at the hospital	1998
Martin 2016	ICD-9 CM	30 harm indicators based on administrative data	Not clear	771
Miano 2019	ICD-9 CM		Electronic medical records	1019
Miller 2016	ICD-9		Not clear	204
Mull 2014	ICD-9 CM	AHRQ PSIs	Electronic medical records—VA EMR system, VistaA	268 771 hospitalizations
Mull 2015	Not clear	AHRQ	Electronic medical records—VA Electronic Health Records	273 cases
Ng 2018	ICD-10AM		Electronic medical records	140
Pellathy 2022	ICD-9 CM		Electronic medical records	3680
Peyton 2019	ICD-9 and ICD-10		Not clear	268
Quan 2013	ICD-10	AHRQ PSIs	Not clear	490
Ramanathan 2013	ICD-9 CM	AHRQ PSIs	Not clear	not clear
Reilly 2020	ICD-10-AM		Electronic medical records	482
Roberts 2008	ICD-10-AM		Not clear	1184 (393 flagged and 791 random)
Rosen 2012	ICD-9 CM	AHRQ PSIs	Electronic medical records—Veterans Health Administration (VA) electronic medical record	1266 cases
Saff 2016	ICD-9 CM		Not clear	1634
Scanlon 2006	Not clear	AHRQ PSIs	Not clear	1132
Scanlon 2008	Not clear	AHRQ PDIs	Not clear	1703
Stacey 2014	ICD-10		Not clear	165
Storesund 2019	ICD-10		Not clear	700 admissions in 695 patient charts
Utter 2009	ICD-9 CM	AHRQ PSI	Electronic and paper-based charts	249
Utter 2010	ICD-9 CM	AHRQ PSI	Electronic and paper-based charts	609 cases
Utter 2013	ICD-9 CM	AHRQ PSI	Not clear	462 (181 indicator positive and 281 indicator negative)
Valentine 2022	ICD-10-AM		Electronic medical records—VERDI Electronic Patient Records system (IPHealth Pty. Ltd.) and the electronic medical record (Epic Systems Corporation,Verona, WI, USA)	151
Valik 2020	ICD-10	Sepsis surveillance based on Sepsis-3-clinical criteria	Electronic medical records	1000 admissions
Verelst 2010	ICD-9 CM		Not clear	1515 (741 flagged for at least one indicator and 774 controls)
Walker 2010	ICD-9 CM	Adverse Outcome Index perinatal quality indicator system that was derived from administrative data	Not clear	342 cases
Walther 2021	ICD-10 GM		Not clear	332
Weingart 2000	Not clear	CSP—a computerized method to identify potentially preventable complications of hospital care using discharge abstract data information	Not clear	1111 cases (831 flagged and 280 unflagged)
Zrelak 2013	ICD-9 CM	AHRQ PSIs	Not clear	324 charts (94 flagged and 230 unflagged)

The medical records system used was unclear in 29 studies as the authors did not specify the format of the charts but referenced using a manual chart review or conducting medical record reviews to validate rates of AEs in administrative data. Paper-based charts, electronic medical records, and a mix of both were used in the remaining studies (Table 3).

## Methodology

### Methodological approaches to chart review

The GTT was used in three studies. A further 7 studies used an unspecified two-stage review approach, and 39 studies used a single-stage review process. The methodological approach used in the remaining seven studies was unclear (Table 2).

### Sampling strategy

The sampling strategies used to identify charts for review are outlined in Table 2. Screening programmes were used in 24 studies to select flagged charts, i.e. charts that had been assigned specific codes, indicating that the patient had experienced an AE. A further 11 studies included both flagged charts and non-flagged charts and the remaining 21 studies selected a random sample of non-flagged charts.

## Level of accuracy identified by validation

### Accuracy

The sensitivity, specificity, PPV, and NPV of the administrative data-based identification of AEs validated against medical records, where found, in addition to the number of AEs detected by chart review and in the administrative data are presented in Table 4. Administrative data were reported to be accurate for detecting AEs in 11 studies; however, two studies [34, 35] suggested that chart review is a poor method of detecting AEs. The administrative data and detection tools did not accurately represent rates of AEs in 31 studies. Administrative data were reported to be useful for monitoring patient safety and identifying potential patient safety incidents but not for public reporting or measuring performance by four studies. A further 10 studies reported varied or moderate accuracy and suggested that administrative data have potential for monitoring AEs.

**Table 4.** Included studies—author, no. of AEs identified by chart review, no. of AEs identified by administrative data, sensitivity, specificity, PPV, NPV, verbatim statement by authors on validity/usability of data, summarized validation outcome.

First author	No. of AEs by chart review	No. of AEs in administrative data	Sensitivity	Specificity	PPV	NPV	Verbatim statement by authors on validity	Summarized validation outcome
Ackroyd-Stolarz	164	135	67%	89%			It is feasible and valid to identify pressure ulcers, fall-related injuries, and adverse drug events in older hospitalized patients using routinely collected administrative hospitalization data	Accurate
Amelung		41			59%		ICD-10-code-based calculations might overestimate patient harm and economic losses	Not accurate
Ammann 2017	34	131			27%		Position-unspecified diagnoses were unlikely to represent true AIS cases. PPVs for principal and secondary inpatient diagnosis codes were higher, but still meaningfully lower than estimates from prior chart validation studies	Not accurate
Ammann 2018	38	75			61%		Our study indicates that principal-position inpatient ICD-9 CM diagnosis codes for DVT and PE recorded in the Sentinel Distributed Database (SDD) have a high PPV (90%) for acute venous thromboembolism (VTE)	Accurate
Awad	544	195					Both claims data and National Surgical Quality Improvement Program (NSQIP) accurately recorded major complications, but were suboptimal compared to chart abstraction in capturing minor complications. Better systems therefore need to be put in place expeditiously to adequately benchmark the outcomes of surgical management of patients with head and neck cancer	Varied accuracy—accurate for major complications but not for minor complications
Bensley	27	18 true-positive, 8 false-positive, 9 false-negative	66.70%	99.40%	69.20%	99.30%	While perioperative stroke rates were similar between administrative data and chart review, administrative data identified the wrong patients and had poor accuracy	Not accurate
Borzecki 2011	75	119			63%		In its present state, PMD should continue to be used as a screen for patient safety events as opposed to a performance measure	Useful for monitoring patient safety but not as a performance measure
Borzecki 2013	25	0 true-positives, 38 false-negative, 68 true-negative					Despite the high PPV of the postoperative respiratory failure (PRF) PSI algorithm, the relatively high proportion of FNs suggests that publicly reported rates should be interpreted with caution. Improved ventilation/intubation coding will have more of an impact on case identification than adding ICD-9 CM codes to the algorithm	Not accurate
Caminiti	91	189 cases	41%	89%	18%	96%	Screening accuracy was 86%. This approach has the potential to allow the timely identification of AEs, enabling to quickly devise interventions. This detection system could be of true benefit for hospitals that intend assessing their AEs	Accurate
Campbell	19.4% (major complication) and 31.6% (minor complication)	17.4% (major complication) and 43.8% (minor complication)					Both the retrospective review and ICD-9 assessment underreported incidence. These findings illustrate another significant weakness and potential source of inaccuracy in the use of population-based ICD-9 and retrospective data capture methodologies	Not accurate
Chen	192 (97 Postoperative Wound Dehiscence (PWD) and 95 Accidental Puncture or Laceration (APL))	224 admissions					Among the 224 flagged medical records (112 per PSI), a total of 97 true PWD cases and 95 true APL cases were identified	Moderate accuracy—potentially useful
Cheng	789	1384			57%		The PPV of ICD-10 CM T codes analysed in our study was insufficient for identifying ADEs during hospitalization. The sensitivity and specificity of this were inadequate	Not accurate
Duke	60 AEs in 462 non-flagged, 423 AEs in 260 flagged		45%	97%	74%	90%	The predictive value of the current HAC methodology for these adverse events was reasonable, but poor at identifying hospital-related or healthcare errors	Not accurate
Eastwood			31.30%	94.60%			As evidenced by low specificity in Hospital #1 coding, coding harms in ICD-11 was particularly challenging	Not accurate
Eisler	240—any aspiration, 9—perioperative aspiration				95%—any aspiration, 3.5%—perioperative aspiration		International Classification of Diseases, Ninth Revision codes for aspiration used as a secondary data source were nonspecific for perioperative aspiration, but when combined with record review yielded a 30% increase in identified cases of aspiration over quality assurance data alone	Not accurate
Foglia	530	502	81.70%	98.20%	86.30%	97.40%	Caution is advised when using the AOI as an exclusive measure of assessing obstetric quality because it may be heavily influenced by a single outcome measure; perineal laceration rates represented twice the frequency of all other outcomes combined. The AOI should be modified to better measure preventable adverse events and include a means of accounting for pre-existing conditions	Not accurate
Fujiwara					PPV ranged 0–100%		For AEs, PPV tended to be low overall with a definition based on ICD-10 alone, suggesting that a combination of definitions based on specific treatment modalities for AEs could be more appropriate	Not accurate
Geraci	263	98	14% (Exclusive)	96%	37% (Exclusive)		In conclusion, we found that ICD-9 CM codes in administrative data were poor measures of in-hospital complication occurrence in our study population. Our results suggest that administrative data should not be used at the present time as a primary source of information on complication rates or provider practice profiles	Not accurate
Graham	24	11 true-positives, 2 false-positives and 13 false-negatives	45.80%	95.80%	84.60%	78%	Administrative data demonstrated a lower sensitivity and measure of agreement with the gold standard	Not accurate
Guzzo	4	45					In conclusion, looking at the objectives of this study, one could argue that the use of administrative data is far from being perfect in a context of patient safety management, and this could be important from the medico-legal point of view	Not accurate
Ho	397	163 (definition 1), 670 (definition 2)	27.7% (definition 1), 32.8% (definition 2)	98.8% (definition 1), 95.9% (definition 2)	91.7% (definition 1), 79.3% (definition 2)	73.9% (definition 1) 74.6% (definition 2)	Discharge abstract database (DAD) underestimate pressure ulcer prevalence. Low sensitivity of DAD for identification of pressure ulcer suggests that this data source may not be accurate for determining overall prevalence, and it should be used with caution if it is being compared with other prevalence studies	Not accurate
Hougland	Flagged sample—704 true Adverse drug events (ADEs), 286 in-hospital ADEs. Random sample—224 inpatient ADEs, 58 ADEs causing admission	Flagged sample—1122. Random sample—23 inpatient ADEs, 32 ADEs causing admission	10% 23/224 (inpatient ADEs—random sample). 55% 32/58 (causing admission—random sample)	97% 1689/1737 (inpatient ADEs random sample). 97% 1855/1903 (causing admission random sample)	63% for any ADE and 25% for inpatient ADE		Flagged ADE codes have an overall PPV of 63% and detect just over half of ADEs causing hospital admission. These codes have a positive predictive value of 25% for inpatient ADEs but detect only 10% of overall inpatient ADEs. Flagged ADE codes provide an imperfect but immediately available ADE surveillance system	Not accurate
Kaafarani	48 post-op pulmonary embolism (PE) or deep vein thrombosis (DVT). 82 iatrogenic pneumothoraces. 95 accidental puncture or laceration	112 for each adverse event (post-op PE/DVT, iatrogenic pneumothoraces, accidental puncture/laceration)			43% for PE or DVT. 73% for iatrogenic pneumothoraces. 85% for accidental puncture or laceration.		Until coding revisions are implemented, these PSIs, especially pPE/DVT, should be used primarily for screening and case-finding. Their utility for public reporting and pay-for-performance needs to be reassessed	Useful for screening and case-finding but not for public reporting or performance
Kandel	586 admission and 354 AEs		88% (92% diagnosis and procedure codes combined)	1	78%	100%	Compared to PJI diagnosis codes, combinations of diagnosis and procedure codes improve the detection of a Prosthetic hip and knee joint infections (PJI) in administrative databases	Accurate
Kuklik 2017	1302 cases	2326					When focusing on specific ADE codes, routine data can be used as markers for npADEs and medication errors, thus providing a meaningful complement to existing drug surveillance systems	Accurate
Kuklik 2019	736 cases had ICD coded diagnosis and 525 cases had ADE confirmed for PPV assessment. 495 ADEs were identified in the sensitivity assessment	186 AEs with no drug-related causation and 60 with drug-related coding. Overall 246 AEs were coded	37.6% for no drug-related causation and 12% for drug related coding. Overall sensitivity was 49.7%		91.20%		Overall, the results confirm the potential of utilizing ICD-10 GM diagnoses coding for ADEs from administrative routine data in hospital monitoring systems	Accurate
Maass	456	171	6–100%	99–100%	33–100%		Indicators based on German administrative data deviate widely from indicators based on clinical data. Therefore, hospitals should be cautious to use indicators based on administrative data for quality assurance. However, some might be useful for case findings and quality improvement	Varied accuracy and hold potential—useful for case finding and quality improvement but not quality assurance
Magnéli	2116 AEs in 1171 patients	1145	5.70%	95.20%			The instrument sensitivity for AEs was very low for both 30 and 90 days, but the specificity was high for both 30 and 90 days. The studied instrument is insufficient for valid measurements of AEs after hip arthroplasty	Not accurate
Martin	297	408	65%	85%	59%	88%	The administrative harm measurement tool (AHMT) is sufficiently accurate for use as a within hospital tool to reliably detect and track harm. Nevertheless, it is not recommended as a tool to make comparisons across institutions, which has policy and payment implications	Useful to track harm but not for public reporting and payment (coding)
Miano	935	815	87.20%	99.60%	91.60%	99.4	We observed higher specificity and lower sensitivity for registry defined VTE compared to administrative data. The low false-positive rate of the registry supports its validity in VTE outcomes research. Further investigation is needed to evaluate the relevance of the variable sensitivity, likely due to definitional differences. Supplementation of registry data with administrative data may strengthen its validity	Accurate
Miller	1122	1202	10.9–99.1%	56.2–99.9%	21.1–98.7%	86–99.9%	The current system of AE reporting for cooperative oncology group clinical trials in paediatric acutemyeloid leukaemia underestimates AE rates	Not accurate
Mull 2014	6005		31–68%	99.1–99.8%	31–72%		These results suggest that the PSIs have moderate criterion validity; however, some surgical PSIs detect different AEs than VASQIP	Moderate accuracy—potentially useful
Mull 2015	109	5 (PSIs), 9 (Veterans Health Administration Quality Improvement Program (VASQIP)), 11 (surgical diagnosis-related group (DRG))					The GTT identified previously undetected AEs at one VA. The GTT has the potential to track AEs and guide quality improvement efforts in conjunction with existing AE surveillance mechanisms	Not accurate
Ng	128	140			91%		CCS is a feasible and reliable approach for the routine monitoring of ADEs in hospitals	Accurate
Pellathy	158	219	55%		40%		ICD-9 CM coding missed diagnostic test-confirmed HA-VTE cases and inaccurately assigned cases without confirmed VTE, suggesting dependence on administrative coding leads to inaccurate HA-VTE phenotyping	Not accurate
Peyton	215 AEs in 122 charts	145 AEs in 80 charts	38.50%	77.40%	58.80%	60.10%	Notwithstanding possible limitations, the level of disagreement of ICD-9 and 10 codes with clinical chart review and the relatively poor performance of code based complication reporting, including underreporting and low sensitivity, show that claims codes are not reliable or complete sources for identifying complications after cystectomy	Not accurate
Quan			12.5–89.5%				Several PSIs had high PPV in the ICD administrative data and are thus powerful tools for true-positive case finding. The tools could be used to identify potential cases from the large volume of admissions for verification through chart reviews. In contrast, their sensitivity has not been well characterized and users of PSIs should be cautious if using them for ‘quality of care reporting’ presenting the rate of PSIs because under-coded data would generate falsely low PSI rates	Moderate accuracy—potentially useful
Ramanathan	321	388			83%		We conclude that the validity of PSIs is low and could be improved by increased education for clinicians and coders. In their current form, PSIs remain suboptimal for widespread use in public reporting and pay-for-performance evaluation	Not accurate
Reilly	558		0–100%	91.6–99.8%	0–80%	78.8–100%	Using International Statistical Classification of Diseases and Related Health Problems (10^th^ edition) Australian Modification codes to identify postoperative complications at our hospital has high specificity but is likely to underestimate the incidence compared to clinical audit	Not accurate
Roberts	192 (188/393 flagged and 4/791 random)		72.9% (flagged cases)	98.5% (random sample)	47.3% (flagged cases)	99.5% (random sample)	PHDS can be used reliably to identify women who suffer a major adverse outcome during the birth admission and have potential for monitoring the quality of obstetric care in a uniform and cost-effective way	Accurate
Rosen	747				28–87%		Overall, PPVs were moderate for most of the PSIs. Implementing POA codes and using more specific ICD-9 CM codes would improve their validity. Our results suggest that additional coding improvements are needed before the PSIs evaluated here in are used for hospital reporting or pay for performance	Moderate accuracy—potentially useful
Saff	444 admissions	1634 admissions					We found that dual coding with a relevant ICD-9 CM code and a relevant E code was found to have a higher likelihood of identifying an allergic drug reaction (76%) compared with coding patients with a possible drug allergy with ICD-9 CM code alone (31%) or an E code alone (12%). Specific ICD-9 CM and E codes can be used in combination to identify allergic drug reactions. Further study of these codes in the inpatient and outpatient settings is necessary to better understand the utility of diagnosis codes for improving epidemiologic research on drug allergy	Not accurate (using codes alone)
Scanlon 2006		1151					The chart review confirmed previous work done with administrative data; the indicators complications of anaesthesia, death in low mortality diagnosis-related groups, and failure to rescue were inaccurate in the paediatric population. Clinician chart reviews to verify paediatric patient safety events suggest that 8 of 11 PSIs are useful for quality improvement in paediatric patients but are inadequate for public comparison of hospital performance	Useful for quality improvement but not for comparison of hospital performance of accountability
Scanlon 2008	1695	1942			PPV for events clearly preventable ranged 0–51.4%. PPV for events clearly/possible preventable ranged 20–80.7%		A subset of paediatric quality indicators derived from administrative data are reasonable screening tools to help hospitals prioritize chart review and subsequent improvement projects. However, in their present form, true preventability of these complications is relatively low; therefore, the indicators are not useful for public hospital comparison	Not accurate
Stacey	146	137					Review of ICD-10-coded AMEs can provide valuable information to improve patient safety and quality	Accurate
Storesund	GTT found 331 complications in 212/700 admissions	519 complications in 332/700 admissions. After excluding codes for complications Present on admission (POA), 298 in 141/700 admissions					Crude data with unverified ICD-10 codes significantly overestimate surgical complications within hospitals because complications present on admission are included	Not accurate
Utter 2009	226	249			91% from a coding perspective but 68% from a clinical perspective of cases that involved consequential injuries		Although PSI 15 is highly predictive of APL from a coding perspective, the indicator is less predictive of APL that could be considered clinically important	Accurate (in terms of coding)
Utter 2010	507	609			59–86%		Although it appears to have high PPV and adequate sensitivity based on a previous study, PSI 11 does not currently appear as promising for its construct validity	Not accurate
Utter 2013	126	181	42%	99.90%	78%	99.70%	Quality indicators based on administrative data carry some degree of inherent inaccuracy as a trade-off for the advantage of capturing information broadly at minimal expense… it seems to hold promise for detecting cases of serious and possibly preventable complications	Moderate accuracy—potentially useful
Valentine	27	151			18%		Although ICD-10-AM data are readily available in Australian healthcare settings, these data are not sufficient for monitoring and reporting of hospital-acquired pneumonia in haematology-oncology patients	Not accurate
Valik	311/674 patient with suspected infection and 2/326 without suspected infection. 343 in total fulfilled sepsis-3 criteria	59					Explicit sepsis ICD-codes seem to underestimate the incidence of sepsis compared to clinical data	Not accurate
Verelst					29.9–62.3%	93.8–99.6%	The B-HDDS has the potential to accurately detect some but not all adverse events. Adding a code ‘present on admission’ and improving the ICD-9 CM codes might already partially improve the correspondence between the B-HDDS and the medical record review	Not accurate
Walker		279			0–100% for 10 indicators		The adverse outcome index (AOI) measures that were based on administrative data were of variable accuracy	Varied accuracy—potentially useful
Walther	308		38.2% for Postpartum hemorrhage (PPH) and 36.2% for severe PPH	99.4% for PPH and 99.4% for severe PPH			Hospital discharge data are not accurate enough to assess the incidence of postpartum haemorrhage at hospital or national level	Not accurate
Weingart	458/831 flagged cases and 21/280 unflagged cases. In total 479 complications identified	831					For some types of complications, screening administrative data may offer an efficient approach for identifying potentially problematic cases for physician review	Moderate accuracy—potentially useful
Zrelak	60 (flagged) and 3 (random)	94	67%	99.90%	64% (60/94 charts with correct coding)	99.9% (3/230 false-negative charts)	PSI 10 mostly concerns AKI and currently has moderate criterion validity, which might improve with increased use of ‘present on admission’ coding, abandonment of the diabetes criteria, and adjustments to the indicator specifications regarding dialysis access and urinary tract obstruction	Moderate accuracy—potentially useful

## Discussion

### Statement of principal findings

This review highlights trends in relation to the characteristics of validation studies and presents an overview of the methodological approaches and strategies used to conduct retrospective chart reviews. It also explores the accuracy of AE rates in administrative data.

### Strengths and limitations

The focus on breadth rather than depth of knowledge is an inherent limitation of scoping reviews [36]; however, this methodology was deemed suitable as it is appropriate for addressing broad research questions and mapping evidence from a variety of sources [37].

### Interpretation within the context of the wider literature

Geographical location of the included studies is notable. Previous researchers similarly identified the USA as a leading location where validation studies are being conducted [10, 21, 38]. The proportion of AHRQ PSI studies included may have contributed to this finding as these indicators are unique to the USA. Collection of administrative data in the USA for billing and insurance claims purposes may be incentive for improving data accuracy and contribute to greater numbers of validation studies. Further global exploration of validating AE rates in administrative data through chart review may allow for broader comparison between health systems.

The upward trend in relation to year of publication demonstrates increasing recognition of administrative data as a source of information on AEs and a momentum towards enhanced accuracy. Retrospective chart review is commonly used to assess quality of care and collect data in clinical epidemiological research [39]. Therefore, it is unsurprising that chart reviews have been used in response to calls for validating administrative data.

The potential of the chart review methodology to validate rates of AEs in various specialties in administrative data was highlighted by this review. Surgical AEs such as wound infections, post-operative complications, and accidental punctures or lacerations were most commonly validated and are among the most reported in-hospital AEs [40, 41]. Although reporting surgical AEs may be associated with litigation and repercussions regarding reputation [42], their disclosure decreases the chances of a patient filing a lawsuit [43–46]. Given the informed consent process and the acknowledgement of such AEs as possible complications, few surgical AEs should be considered truly unanticipated [47]; therefore, they are more likely to be readily acknowledged than AEs resulting from negligence or medical error. As surgical AEs are more visible, their detection may be more common than AEs in other specialties with 97% of surgeons reporting that they would definitely disclose such an AE to a patient [48]. The PSIs are largely composed of surgical indicators [49]; therefore, their inclusion may have contributed to the focus on surgical AEs. Given the burden of surgical outcomes in relation to reputation and litigation, accurate data and a standardized chart review method would be beneficial to surgeons for monitoring AEs.

The various named study designs identified reiterate the lack of standards for reporting chart review study designs and the limited availability of literature on chart review methodologies [50]. Evidently, there has been little progress towards developing a more systematic and standardized review approach. This further highlights the urgency for consensus on best practice for conducting chart reviews.

As this study sought to explore manual chart review methods, sources using Artificial Intelligence (AI) were excluded; however, when discussing study designs, the increasing use of AI is notable [51–56].

AEs detected through incident reporting systems have recently been compared to those detected using the GTT [57]. This current review explores all methodological chart review approaches used to validate rates of AEs in administrative data. In line with previous research [26], the variation of single-stage and two-stage chart review processes demonstrates a lack of consensus on how to measure AEs. A two-stage review process has been identified as a valid and reliable method of conducting chart reviews [58]. The HMPS and GTT have frequently been compared with some researchers identifying higher sensitivity and specificity and better AE detection using the GTT [59] and others identifying more AEs using the HMPS [60]. This further demonstrates the lack of consensus in relation to guidelines for best practice.

The strategy for obtaining a chart review sample is a key consideration [23]. Random sampling is the gold standard as each chart has equal chance of being selected. This technique reduces bias and increases the generalizability of results [23] and should be considered when developing a more systematic chart review approach. Reviewing a random sample of charts allows for detection of all potential AEs and inclusion of false-negatives missed by detection tools if administrative data are inaccurate. A limitation of this method is the large number of medical records required which may not be possible in all chart reviews [23].

A random process for selecting records is critical when using the GTT [61]. The included GTT studies used random sampling; however, the use of flagged charts was widespread across other methodological approaches. This further enhances the need for consensus in relation to the chart review method and the strategies used to identify samples. Internal and external validity may be compromised if studies fail to use a random sample or if a sample is selected from an atypical population [62].

Convenience sampling allows charts identified as having an AE by administrative data-based detected tools to be selected. This validates known cases of AEs but may exclude miscoded or unassigned AEs missed by detection tools and underrepresent true AE rates. This method presents limitations in relation to generalizability but is useful when reviewing rare AEs or smaller sample sizes [23].

Reviewing only flagged charts was identified by several authors as a limitation [63–68] as excluding non-flagged charts may have underrepresented AE incidence by omitting false-negatives [63, 68]. The value of these studies is worth considering as rather than validating administrative data, they explored the validity of indicators used to flag the sample of charts as measures of patient safety [16]. It may be more valuable to assess the validity of the coding to ensure that the data being screened are accurate.

Including both flagged and non-flagged charts allows true-negatives and false-negatives to be detected and ensures that AE rates are not underestimated [63]. Furthermore, flagged and random samples are advantageous in research investigating rare conditions as an entirely random sample may be inefficient and resource demanding [69].

The range of ICD revisions and variants used contributes to the complexity of comparing coded data and clinical contexts internationally [70]. The studies that reported on the accuracy of administrative data-based detection tools relied on different modifications and revisions of ICD to create their patient safety data. Therefore, the range of ICD revisions and systems used must be considered as a potential source of result variation. Comparability of AEs internationally is threatened by country-specific modifications of ICD; therefore, a standardized ICD classification that is universally accepted may facilitate benchmarking of AEs using administrative data [9].

Furthermore, the medical record system used and the number of charts reviewed are important features of the studies in this review. Parallel use of both paper-based and electronic medical records may result in inconsistencies between record systems [71] and may have contributed to variation in results. Consequently, researchers may wish to consider the type of medical record system used in future validation studies. Standardized processes for entering data and limiting variation in medical records increase the accessibility and usability of chart data. Furthermore, the development of consistent data collection procedures and more systematic approaches are critical to rigour in chart review studies [72].

Sample size is a key sampling consideration in chart review studies. Greater power is associated with studies that have larger sample sizes [23]. The number of charts reviewed may be valuable to researchers planning future studies as chart reviewing can be time-consuming and the feasibility of locating charts and extracting data may require consideration [73].

Although various levels of accuracy were identified, administrative data can potentially provide accurate information. In line with previous research [28], while some studies indicated that these datasets can be reliable, others identified inaccuracies when comparing administrative data with chart data. The studies that identified rates of AEs recorded in administrative data with good accuracy [69, 74–83] strengthen the case for using administrative data to inform health research and policy. Although further work is warranted, these findings are in agreement with previous claims that with consensus on rigorous methodology for validating and improving existing algorithms, administrative data can provide valuable information on AE rates and patient safety incidents [28].

### Implications for policy, practice, and research

This review highlights the potential for administrative data to improve patient safety, healthcare quality, and reduce healthcare costs by providing valuable data on AE rates; however, further research is warranted to ensure that administrative data are robust. The lack of consensus on best practice for conducting chart reviews is highlighted. The different methodological approaches and sampling strategies used demonstrate the potential for these studies to differ significantly in relation to the interpretation of their results and their credibility. These inconsistencies mirror previously identified common pitfalls [23] and may devalue the chart review method of validating rates of AEs in administrative data. The development of a standardized protocol with clear guidelines could enhance the methodological rigour of the retrospective chart review and allow for the accuracy of administrative data to be improved.

## Conclusions

Accurate administrative data will enable researchers to compare hospital environments in the context of patient safety and facilitate benchmarking of AE rates across hospitals, regions, and countries. Further clarity and consensus on chart review methods is necessary to develop a more systematic chart review approach for improving patient safety and healthcare quality.

## Supplementary Material

mzae037_Supp

## Data Availability

No new data were generated or analysed in support of this review. As this is a scoping review, all data have been presented within the article and its online supplementary material.
