# Supplementary material for: A scoping review of the methodological approaches used in retrospective chart reviews to validate adverse event rates in administrative data
Source: Int J Qual Health Care. 2024 Apr 24;36(2):mzae037. doi: 10.1093/intqhc/mzae037 (PMC11086704; doi:10.1093/intqhc/mzae037)
Supplement: mzae037_Supp [file mzae037_supp.zip › suppl_data/Supplementary Material - Search Strategy.docx]

Supplementary material

Search Strategy for identifying sources of evidence for inclusion in the scoping review

| Search | Search Terms |
| --- | --- |
| S1 | “discharge data” OR "hospital discharge data" OR “routinely collected data” OR "routinely collected discharge data" OR “administrative data” OR "administrative health data" OR "healthcare administrative data" |
| S2 | verif* OR valid* OR compar* OR evaluat* |
| S3 | S1 AND S2 |
| S4 | "chart review" OR "record review" OR "medical record review" OR "clinical notes" OR "retrospective chart review" |
| S5 | S3 AND S4 |
| S6 | "adverse event*" OR "adverse outcome*" OR "healthcare acquired complication*" OR "patient safety incident*" |
| S7 | S5 AND S6 |
